# Supplementary material for: Are carbon emissions trading and green financial instruments synergistic? -Comprehensive quantitative research based on content analysis
Source: PLoS One. 2024 Mar 7;19(3):e0298601. doi: 10.1371/journal.pone.0298601 (PMC10919601; doi:10.1371/journal.pone.0298601)
Supplement: S1 Data — (ZIP) [file pone.0298601.s001.zip › Data/地级市Z_F工作报告.zip!/║╙─╧/╓ú╓▌╩╨/└┤╫╘╣1⁄2╓┌║┼í╛┬φ┐╦╩2╛▌═°í┐_2016.docx]

各位代表：

现在，我代表市人民政府，向大会报告工作，请予审议，并请各位政协委员和列席人员提出意见。

一、“十二五”成就及“十三五”主要目标

“十二五”时期，是我市发展史上极不平凡的五年。面对复杂多变的外部环境和经济下行压力持续加大的严峻形势，在省委、省政府和市委的坚强领导下，全市上下贯彻落实“四个全面”战略布局，按照“全国找坐标、中部求超越、河南挑大梁”的总要求，抢抓航空港实验区和中原经济区建设重大机遇，围绕“三大一中”战略定位，突出“三大主体”工作，大力实施开放创新双驱动战略，积极作为经济发展新常态，干成了一系列打基础利长远的大事要事，经济社会持续健康快速发展，顺利完成了“十二五”的各项目标任务，全面建成小康社会站在了新的历史起点。

——综合实力迈上新台阶。“十二五”时期，我市保持了年均11.2%的经济增速，高于全国、全省3.4个和1.6个百分点，各项主要经济指标在全国、全省保持了升级晋位态势。2015年，全市生产总值完成7315.2亿元，总量比2010年增长70%，占全省比重由17.5%提高到19.8%，对全省增量的贡献率达到26%，在全国27个省会城市中由第8位前移至第7位；一般公共预算收入942.9亿元，是2010年的2.4倍，占全省比重由28%提高到31.5%，对全省增量的贡献率达到40.3%，在全国27个省会城市中由第7位前移至第6位。郑州在全省的首位度持续提升，区域竞争力显著增强。

——改革创新步伐明显加快。通过持续深化重点领域关键环节改革，增创区域发展新优势。以行政审批制度改革、“五单一网”制度改革、产业集聚区管理机构套合改革等为标志，着力构建“小政府、大服务”的政务服务新格局；以投融资体制改革、城乡规划土地建设管理“六统一”制度改革等为标志，政府性资源市场化配置能力进一步提升；以创新创业综合体建设、“两金一扶”政策体系和“智汇郑州·1125聚才计划”为支撑，营造了有利于人才集聚、创新创业的发展环境。郑州经济社会发展的内生动力和活力持续增强。

——经济结构不断优化升级。三次产业结构由2010年的3.1︰54.5︰42.4调整为2.1︰49.5︰48.4，高新技术产业增加值在生产总值中的比重由16.5%提高到24%，工业中战略性新兴产业比重由36.5%提高到49.4%，实现服务业在经济中的比重超过工业、工业中战略性新兴产业比重超过传统资源高耗能产业两大历史性转型，经济发展的质量和效益显著提升。

——城乡面貌发生质的飞跃。米字形快速铁路网建设全面展开；地铁1号线开通运营，1号线二期、2号线一期、5号线、3号线一期加快建设；14个环城高速出入口、266条支线路网建成投用；“两环三十一放射”、10条市域快速通道等一大批交通道路工程建成通车，“井字+环线”快速路网初步成型。市区85%的区域用上南水北调优质水源；新增天然气用户60万户，新增供热面积5588万平方米，生活垃圾无害化处理率由90%提高到95%，污水日处理能力由90万吨提高到170万吨，城市承载能力持续增强。全市1994个行政村已有1107个被纳入“四类社区”建设，回迁群众100万人。市县两级城市建成区面积5年增加220平方公里，城镇化率由63.6%提高到69.9%。自然之美、社会公正、城乡和谐的现代田园城市风貌初步展现。

——开放平台支撑更加坚实。航空港实验区上升为国家战略，实验区建设取得重大阶段性成果，“三年打基础”目标基本实现。机场二期建成投用，货邮吞吐量增速连续4年居全国大型机场前列。综保区等海关特殊监管区功能不断拓展，汽车、肉类等进口口岸投用，我市成为内陆功能性口岸最多的城市。郑欧班列境内外集疏分拨范围、货运总量和满载率均居中欧班列前列。跨境贸易电子商务走货量居全国试点城市前列。富士康、国家专利审协河南中心等一大批国内外知名企业和国家级功能平台落户郑州。郑州成为国家级互联网骨干直联点，跻身全国十大通信网络交换枢纽。全市累计利用境外投资167.3亿美元，是“十一五”的2.6倍；进出口总额由51.6亿美元增长到570.3亿美元，占全省76.7%，年均增长61.6%，在全国27个省会城市中由第17位跃升至第3位。特别是圆满完成了上合组织成员国政府首脑理事会第14次会议服务保障任务，充分展示了我市的整体形象和实力。郑州在全国、全省发展大局中的地位、作用和影响力明显提升。

——群众生活水平持续改善。全市公共财政民生支出2889亿元，年均增长26%。城镇就业人数净增72万。新建改扩建中小学校165所、幼儿园416所。“五险合一”市级统筹基本实现全覆盖，城镇基本养老保险参保率95%以上，新农合参合率99.5%。346万农村人口饮水安全问题得到解决，在全省率先实现了农村饮水安全村村通自来水目标。精准扶贫成效显著，全市24.5万扶贫对象已有20.6万人实现脱贫。城镇居民人均可支配收入由1.9万元提高到3.1万元，农村居民人均可支配收入由9225元提高到1.7万元，年均分别增长10.5%、13.2%。

这些成绩的取得，是落实省委、省政府决策部署的结果，是市委正确领导的结果，是市人大依法监督、市政协民主监督的结果，是全市上下开拓创新、务实重干的结果。在此，我代表市人民政府，向辛勤奋战在各条战线上的广大干部群众、驻郑人民解放军、武警官兵、政法干警和中央驻郑单位，向各民主党派、工商联、各人民团体和社会各界人士，向所有关心、支持郑州改革开放与现代化建设的海内外朋友表示诚挚的感谢和崇高的敬意！

经过“十二五”时期的探索实践，我们积累了宝贵经验，主要体现在：坚持科学发展。把贯彻中央、省委决策部署与郑州实际相结合，把握大势，遵循规律，坚持走好以“一个载体、三个体系”为支撑的“三化协调、四化同步”科学发展路子。坚持开放带动。充分发挥区位加交通比较优势，抢占先机，主动作为，着力打造开放载体平台，建设大枢纽，发展大物流，培育大产业，塑造大都市，积极构建内陆开放高地。坚持改革创新。突出问题导向、市场导向、需求导向，强力推进重点领域和关键环节改革，不断推动体制机制创新，以“双创”为带动，持续增强发展的内生动力和活力。坚持加快发展方式转变。紧紧围绕建设资源节约型和环境友好型社会，突出集约集群集聚发展，着力促进结构调整和经济转型，加快构建现代产业体系，不断提高城市核心竞争力。坚持统筹城乡协调发展。大力推进以人为核心的新型城镇化，努力形成合理的城镇体系、合理的人口布局、合理的产业布局和合理的就业结构，促进基本公共服务均等化，加快城乡一体化进程。坚持共享发展。以办好民生实事为重要抓手，着力在保障改善民生上做文章、在服务质量和服务方式上求提升，以改善民生凝聚民心，以凝聚民心汇聚民力，以汇聚民力共促发展。这些经验是全市人民智慧的结晶，弥足珍贵，必须倍加珍惜、发扬光大。

各位代表：“十三五”时期是我市率先全面建成小康社会的决胜期，也是“抢抓机遇、奠定基础、确立地位”的攻坚期。我们必须以高度的历史责任感、时代紧迫感，准确把握形势的新变化新特点，努力开创科学发展新局面。

《郑州市国民经济和社会发展第十三个五年规划纲要（草案）》、《郑州建设国际商都发展战略规划纲要（草案）》及说明已印发大会，提请审议。

“十三五”时期经济社会发展的指导思想是：高举中国特色社会主义伟大旗帜，全面贯彻党的十八大和十八届三中、四中、五中全会精神，以马列主义、毛泽东思想、邓小平理论、“三个代表”重要思想、科学发展观为指导，深入贯彻习近平总书记系列重要讲话精神，落实“四个全面”战略布局，坚持发展第一要务，贯彻“创新、协调、绿色、开放、共享”发展理念，抢抓航空港实验区和中原经济区建设重大机遇，深入实施开放创新双驱动战略，以国际商都建设为统揽，以新型城镇化为载体，以中高端现代产业体系为支撑，确立郑州国际综合交通枢纽和物流中心、“一带一路”核心节点城市、全球智能终端（手机）制造基地 “三个地位”，在全省率先全面建成小康社会、率先开启现代化建设新征程。

主要预期目标是：经济保持中高速增长，主要经济指标和人均指标增速高于全省平均水平1个百分点以上，力争到2017年率先全面建成小康社会。到2020年，部分区域和领域初步实现现代化，综合竞争力跨入国内城市第一方阵，转型升级和创新驱动实现新突破，产业发展水平迈向中高端，质量、效益和协调性明显高于“十二五”；生态环境质量总体改善，社会治理能力和城市软实力明显增强，人民群众的获得感、幸福感和城市归属感稳步提升，实现国际商都规划阶段性目标。

实现上述目标，我们必须以国际商都建设为统揽，强化国际化、智慧化引领，着力构建“十大体系”，即国际化现代化立体综合交通大枢纽体系，“买全球、卖全球”的大物流体系，智慧化、国际化、高端化的大产业支撑体系，统筹城乡协调发展的大都市城镇体系，与国际接轨、国内领先的大开放体系，全民创业链、科技创新链、人才支撑链“三链融合”的大创新体系，自然之美、田园风貌、绿色低碳的大生态体系，以人为本、共建共享的大民生保障体系，具有国内外影响力、亲和力、感染力的大文化体系，高质高效、公平正义、责任有序的大服务体系。

各位代表：我们坚信，只要全市人民携起手来，凝神聚力，砥砺前行，坚持“五大发展理念”不动摇，矢志不渝促发展，按照“十三五”总体谋划，一件一件抓落实，咬定青山不放松，我们的目标就一定能够实现！

二、2015年工作回顾

2015年是“十二五”的收官之年。一年来，按照市委决策部署，我们坚持抓改革创新、强投资开放、促结构转型、求民生改善，较好地完成了市十四届人大二次会议确定的目标任务。初步统计，全市生产总值增长10.1%，分别高出全国、全省3.2个、1.8个百分点；一般公共预算收入增长13.1%，分别高出全国、全省4.7个、3.2个百分点；固定资产投资增长19.6%，分别高出全国、全省9.6个、3.1个百分点；规模以上工业增加值增长10.2%，增速居全国35个大中城市第4位；进出口总额增长22.9%，增速居全国35个大中城市首位；社会消费品零售总额增长11.5%；城镇居民人均可支配收入增长8.7%；农村居民人均可支配收入增长8.9%。

（一）抓住关键求突破，航空港实验区建设取得重大进展。坚持以体制机制创新、产业集聚、大枢纽建设为抓手，坚定不移打好实验区建设攻坚战。全面推进体制机制创新。复制上海自贸区海关制度创新11项、检验检疫制度创新8项；在全省率先推行了“三证合一”、电子营业执照登记管理等创新改革。加快推进主导产业集聚发展。智能手机产量突破2亿部，约占全球供货量的七分之一；富士康液晶面板等重大项目开工建设，友嘉精密机械产业园等项目加快推进。强力推进大枢纽建设。郑徐高铁主体完工，郑万、郑合高铁开工建设；郑焦、郑机城际铁路通车运营。郑州机场已开通客货航线171条，客运量1729.7万人次，货运吞吐量40.3万吨。多式联运海关监管中心一期建成运营，“四港一体”合作发展机制初步确立。大力推进航空大都市建设。空港、古城、双鹤湖、会展物流“四大片区”联动发展。成功举办2015郑州航展，通用航空在国内外的影响力不断提升。

（二）深化改革激活力，重点领域改革取得新成效。进一步深化重点领域改革，发展内生动力不断增强。持续深化“五单一网”制度改革。公布实施“五个清单”，明确市本级权责7478项、调整行政审批147项、压减行政事业性收费275项，政府性基金压减至11项、确认企业投资项目管理负面清单106项，全市四级政务服务网启动运行，便捷、高效的政务服务环境正在形成。持续增强市场主体活力。加快商事制度改革，市场主体、注册资金分别增长21.9%、15.8%。以“共保体”模式支持科技型、创新型、创业型小微企业发展，贷款余额2455亿元、增长32%；新增挂牌企业50家，挂牌上市公司累计118家，居中部六省会城市前列。持续提升政府性资源市场化配置能力。不断深化经营性土地网上招拍挂和土地统收统储统供新模式，加快存量闲置用地清理处置，盘活土地13万亩；以融资工具和模式创新为重点，多层次、多领域、多渠道开发利用资本市场，累计融资804亿元。

（三）统筹城乡强支撑，新型城镇化建设提质增效。以人的城镇化为核心，新型城镇化迈入全面推进的新阶段。强力推进“畅通郑州”工程。南四环至机场城郊铁路工程顺利推进。未来路下穿金水路等工程竣工通车，南三环东延二期、国道107辅道快速化等工程加快建设。完成支线路网138条，市区新增公共停车泊位8.3万个。加快大棚户区改造和新型农村社区建设。全年累计启动村庄拆迁改造359个，开工建设安置房4414万平方米，回迁群众42.6万人，基本实现围合区域内及县城、产业集聚区、组团新区规划区范围内村庄拆迁改造大头落地；市场外迁58个，三年177个市场外迁的目标基本完成；启动建设新型农村社区156个，建设安置房1177万平方米，回迁群众19.9万人。持续提升中心城区功能。一大批水电气暖等基础设施加快建设或建成投用，以“三级三类”便民服务中心为依托的公共服务体系加快构建，城市精细化管理扎实开展，城市管理水平明显提升。

（四）优化结构促转型，产业结构调整步伐加快。坚持扩大优质增量、调整优化存量并举，推动产业结构转型升级。加快推进工业结构优化。汽车及装备制造、电子信息、新材料、生物医药四大战略性产业占全市工业比重提升了2.6个百分点，传统高耗能产业比重降低了2个百分点。七大主导产业投资占工业投资的比重达到78%，千亿级主导产业达到5个。大力发展现代服务业。加快发展以E贸易、华南城等为带动的新型商贸产业集群，以航空物流、大宗商品交易市场等为带动的现代物流产业集群，以郑州国际文化创意园区等为带动的文化创意旅游产业集群；郑东新区金融集聚核心功能区入驻金融机构达到265家，金融业增加值占现代服务业的比重超过20%。加快发展都市生态农业。粮食产量达到168万吨，连续13年喜获丰收，建成农业产业化集群28个，农产品加工业年销售收入2000亿元。持续加快产业集聚区发展。产业集聚区对工业增长、投资拉动的贡献率分别达到66.7%、47.4%；经开区成为全省唯一的六星级产业集聚区，航空港区成为全省五星级产业集聚区。强力推进重点项目建设。新开工重点项目234个、竣工70个；省市重点项目完成投资4075亿元，完成年度目标的113%，投资率、审批率均居全省前列。

（五）开放创新双驱动，厚植发展新优势。大力实施开放创新双驱动战略，以开放创新促转型、促发展。着力打造开放载体平台。出口加工区B区通过国家预验收，中国（河南）自贸区、经开综保区申建工作顺利推进；郑欧班列全年开行156班，实现了每周“去三返二”的常态化运营；国际邮件转运口岸及肉类、活牛、冰鲜水产品、食用水生动物等进口指定口岸获准运营。加快培育新业态新模式。郑州跨境电子商务综合试验区获国家批准；国家电子商务示范城市建设和国家服务业综合改革试点工作加快推进；跨境贸易电子商务试点业务迅猛增长，走货量全年突破5000万单。持续开展大招商。围绕“四力”型项目引进，强化“五职招商”责任制，实际利用外资38.3亿美元。不断提升自主创新能力。20个创新创业综合体建成110万平方米，入驻企业1060家，培育科技创新团队36个，引进科技领军人才82名。

（六）多措并举抓治理，生态文明建设持续加强。坚持发展、保护两手抓，强力推进生态建设和环境治理。大力实施蓝天工程。拆改市区燃煤锅炉110蒸吨，市区燃煤锅炉拆改任务基本完成；淘汰黄标车和老旧车辆9.7万辆；中心城区机械化清扫率达到80%，建筑工地扬尘治理基本达到“六个100%”整治标准。大力实施生态绿化工程。新增绿地1201万平方米，新建公园游园28个，建成区绿地率达到35.3%；建成森林公园9个、林业生态廊道467公里，植树造林7.49万亩，森林覆盖率达到33.4%。大力实施碧水工程。环城生态水系循环工程、石佛沉砂池向西区供水工程顺利开工，贾鲁河生态水系综合整治工程有序推进。扎实推进农村人居环境综合整治和美丽乡村建设。新增省级人居环境整治达标村（社区）100个、省级示范村（社区）50个、国家级生态乡镇3个。

（七）保障民生增福祉，社会事业全面进步。持续加大民生投入，圆满完成民生实事。创业就业成效显著。发放小额担保贷款10.6亿元，新增城镇就业15万人，农村劳动力转移就业10万人。教育资源配置不断优化。市区新建改扩建中小学校37所、新建幼儿园47所，妥善安置5.6万进城务工人员随迁子女入学。公共卫生服务水平持续提升。一大批优质医疗资源项目投入使用，新增床位9300张；持续完善“片医”基层卫生服务体系，成为全国推进医疗卫生改革示范城市。文化体育事业繁荣发展。深化国家公共文化服务体系示范区创建工作，四级公共文化基础设施网络基本建立，市民公共文化服务区“四个中心”等一大批重大项目开工，乙未年黄帝故里拜祖大典成功举办。社会保障体系不断完善。医保、低保、居民基本养老金、企业退休人员养老金、优抚对象抚恤标准稳步提高；新开工公租房1万套，建成保障性住房4.85万套。慈善事业加快发展，蝉联全国“七星级慈善城市”。精准扶贫加快推进。易地扶贫搬迁1.3万户5万多人、整村推进30个，脱贫群众5.46万人。

（八）转变作风提效能，政府自身建设不断加强。扎实开展“三严三实”专题教育，严格执行中央八项规定及省委、市委有关规定，全市会议费同比下降18%，“三公”经费同比降低7.2%。办理省人大代表建议21件、省政协委员提案44件，市人大代表议案6件、建议591件，市政协委员提案821件，议案、建议和提案全部办结，满意率99.3%以上。向市人大常委会报备政府规章4件、其他规范性文件67件。严格落实“一岗双责”，坚持依法行政，加大效能监察和反腐败工作力度，一批违法违纪人员受到惩处。深化规范提升网格化管理，积极推进平安郑州建设，持续加强非法集资防范处置，狠抓安全生产、食品药品、消防安全等重点领域安全监管，社会大局和谐稳定。

国防教育、国防后备力量建设和“双拥”共建工作取得新成效。外事侨务、对台工作进一步加强。民族团结进步事业取得新进展。统计、审计、档案管理、修志编鉴、社科研究工作水平实现新提升。人防、气象、防震减灾体系不断完善。

在肯定成绩的同时，我们也清醒地认识到发展中还存在诸多矛盾和问题，主要是：产业结构性问题依然突出，传统产业改造升级力度不够，新兴产业拉动能力有待提升；创新基础和科技创新能力相对薄弱，高层次人才依然缺乏；城市综合承载力亟待提升，城市交通、公共服务、精细化管理等方面与群众期盼还有不小差距；生态环境约束加剧，大气、水体、土壤、农村环境等污染问题依然突出；政府职能转变还不到位，一些干部不作为、不会为、不敢为的问题依然存在。对此，我们一定高度重视，直面矛盾和问题，采取有力措施，不断加以改进和解决，努力让人民群众满意。

今年是实施国际商都规划和“十三五”规划的开局之年，也是推进供给侧结构性改革的攻坚之年。世界经济仍在深度调整，总体上延续疲弱复苏态势；我国经济长期向好的基本面没有改变，但结构性矛盾突出，经济下行压力较大。我市与全国一样，形势比较严峻，环境复杂，任务艰巨。同时，我们也要看到，我市多重机遇叠加汇集。以航空运输为主导的“第五冲击波”、互联网、自由贸易时代到来和我国国际地位显著提升，为郑州加速融入全球经济体系提供了重要机遇；郑州成为“一带一路”核心节点城市，国际商都规划上升为全省战略；特别是郑州枢纽优势、开放优势、体制优势逐步显现，大发展的态势和能量正在形成。我们既要正视问题、防范风险、迎难而上，又要抢抓机遇、保持定力、坚定信心，确保经济社会持续健康较快发展。

政府工作的总体要求是：全面贯彻中央、省委、市委各项决策部署，坚持“四个全面”战略布局，牢固树立“创新、协调、绿色、开放、共享”发展理念，主动融入“一带一路”倡议，以国际商都建设为统揽，紧紧围绕“三大一中”战略定位，着力加强供给侧结构性改革，大力实施开放创新双驱动战略，持续提升“三大主体”工作，突出“大开放、大创新、大建设、大管理”，努力实现“十三五”良好开局，为在全省率先全面建成小康社会、率先开启现代化建设新征程奠定坚实基础。

主要预期目标是：地区生产总值增长9%；规模以上工业增加值增长10%以上；固定资产投资增长16%；一般公共预算收入增长9%；社会消费品零售总额增长11%以上；进出口总额增长7%；实际利用外资增长3%；城乡居民收入增长与经济增长基本同步；居民消费价格涨幅控制在3%左右；节能减排降碳、环保等约束性指标完成省下达任务。

今年在统筹做好各项工作的同时，将重点抓好以下工作：

（一）坚持开放带动主战略，着力形成全方位、多层次、宽领域对外开放新格局。以国际化为引领，不断拓展开放的广度深度，持续增强内外联动发展能力。加快航空港实验区建设。以建设航空、铁路、公路“三网融合”现代立体综合交通枢纽为基础，重点提升“四港一体、多式联运”物流功能，统筹推进区域联动发展，协同推进产业集聚和城市功能完善，打造全省体制机制创新示范区，增强国际影响力和带动力。大力推进改革创新和自贸区政策复制，不断完善以自贸区为标杆的“大通关”体系。加快打造国际航空货运枢纽，力争国际货运航线达到36条。加快郑州—卢森堡航空物流双枢纽建设。在改造提升的基础上，逐步形成以上街机场为核心的通用航空运营网络。持续深化与富士康战略合作，加快推进正威智能手机产业园、中国移动河南数据中心等项目建设，力争年产手机2.5亿部。确保生产总值增长15%、规模以上工业增加值增长16%、固定资产投资增长18%。加快开放载体平台建设。争取中国（河南）自贸区近期获批，确保新郑综保区拓展和经开综保区申建顺利完成。加快郑州—波兰铁路物流双枢纽建设，积极开拓郑欧班列增值服务，确保全年开行200班以上。大力发展复合型多式联运，谋划实施国际陆港二期，力争完成货运吞吐量12万标箱。加快跨境电子商务综合试验区建设，力争全年走货量突破1亿单。加强口岸体系建设，实现汽车、邮政、肉类、活牛、水果、冰鲜水产品、食用水生动物等口岸规模化运营，粮食、药品口岸建成运营；推动邮政集团加快对接万国邮联，构建辐射中西部的快件物流中心。突出抓好精准招商。坚持集群式招商、产业链式承接，围绕“四力”型项目和新技术新业态新模式招商，力争引进“四力”型项目50个、实际到位资金1900亿元。

（二）坚持创新引领，加快发展动力转换。把创新放在发展全局的核心位置，加快推进“全民创业热土、国家创新中心”建设，着力构建大都市战略支撑产业体系。

在创新方面。加快推进国家自主创新示范区建设。重点在科技合作、科技成果转化、高端人才引进等方面先行先试。加快“双创”支撑平台建设。积极推进中科院过程研究所郑州分所等项目建设，新增国家级、省级科技创新型孵化器10家以上；确保政府主导的20个创新创业综合体建成投用120万平方米、新入驻企业1500家以上，力争年底前15个创新创业综合体实现科技公共服务平台全覆盖。加快实施“智汇郑州·1125聚才计划”。力争年内引进培育高端人才和紧缺型高层次人才200名、领军型科技创新创业团队20个。全面贯彻落实网络强国战略。加快推进智慧城市、宽带中国示范城市及下一代互联网示范城市建设；深入贯彻国家大数据战略，促进互联网与交通、医疗、行政服务等领域深度融合；以互联网促进三次产业转型升级，形成拉动经济增长的新动力。

在结构调整方面。以加快新旧动力转换为核心，大力推进技术改造和新产品研发，推动郑州制造向郑州创造、郑州速度向郑州质量、郑州产品向郑州品牌转变。突出高端引领，做大做强战略性产业。以智能终端产业为引领，加快液晶面板、智能穿戴等相关产业发展，着力打造全球重要的智能终端产业基地、国内领先的电子信息产业基地，智能终端产业销售收入突破3000亿元，电子信息产业规模力争达到3600亿元。以汽车及高端装备制造为引领，加快推进东风日产第二工厂、中国中车轨道交通基地等项目建设，力争新能源汽车生产能力达到6万台、整车产能达到100万辆、汽车及装备制造业销售收入突破3000亿元。以新材料和生物医药产业为引领，加快发展战略性新兴产业，力争新材料产业销售收入突破2000亿元。加快提升改造传统优势产业，推动现代食品、铝精深加工、品牌服装及现代家居制造三个千亿级产业基地建设。突出需求导向，促进服务业提质增效。大力发展消费金融、金融租赁等新兴业态，强力推进郑东新区金融集聚核心功能区和龙湖金融中心建设；加快国家信息消费试点城市建设，扎实做好国家内贸流通体制改革试点工作，积极申报国家服务外包示范城市；提升电子商务产业规模和聚集程度，力争电子商务交易额达到4500亿元、增长25%。以民生需求为导向，促进教育培训、健康养老、文化体育、家政服务等生活性服务业加快发展。以华强文化科技产业基地等一批重大项目建设为带动，整合旅游资源，大力发展全域旅游。支持登封市建设以华夏历史文明传承创新示范工程为主导的世界历史文化旅游名城。突出转型融合，发展都市生态农业。加快转变农业发展方式，推进一、二、三产业融合发展，抢占农业制高点，建成高标准农田4万亩、可追溯标准化“菜篮子”生产基地3万亩、都市生态农业示范园6万亩。突出集约集聚，推进产业集聚区晋星晋级。持续推进产业集聚区上规模上水平上层次，加快服务业“两区”提速扩容，形成营业收入超百亿元产业集群6个。

（三）坚持深化重点领域和关键环节改革，持续增强发展活力。以处理好政府和市场关系为核心，各项改革任务、制度建设向全面建成小康社会目标聚焦、向构建发展新体制聚焦，确保改革取得预期成效。扎实推进供给侧结构性改革。积极稳妥化解过剩产能，通过发挥市场在资源配置中的决定性作用，促进过剩产能优胜劣汰、重组兼并、嫁接提升，实现以增量带存量，推动产业转型升级。大力去除商品房库存，以县（市）区为主体，以市场为导向，研究制定各区域商品房去库存、促消费具体措施；探索租购并举的住房制度，从今年起原则上不再新建公租房，新增公租房需求主要通过发放货币补贴、由政府购买或租赁存量商品房解决；完善大棚户区改造安置政策，提高货币化安置率。全面落实国家、省降低企业税费负担、社会保险费、电力价格等政策，研究制定降成本优供给推进发展政策措施，帮助企业降低生产经营成本，减轻企业负担，推动实体经济持续健康快速发展。深化经济体制改革。以投融资体制改革、金融支持小微企业改革、财税体制改革为支撑，以商事制度改革、营改增扩围、消费税改革和资源税费改革为突破，持续提升实体经济发展活力。力争小微企业融资余额达到2800亿元，新增资本市场融资600亿元。深化资源要素市场化配置。大力发展城区经济和县域经济；持续推进国有企业改革，提高国有企业核心竞争力和国有资本效率；鼓励支持引导非公有制经济发展；改进政府调控方式，放开竞争性领域商品和服务价格。充分发挥资本市场作用。设立50亿元规模的PPP项目基金，争取撬动社会资本100亿元以上；积极向上争取债务置换额度380亿元以上，争取年内债券融资规模达到200亿元以上；积极推动企业挂牌上市，力争年内新增挂牌上市企业30家以上；开展投贷联动试点，对创新创业和战略性新兴产业发展提供融资支持。

（四）坚持科学推进新型城镇化，加快打造和谐宜居、富有活力、独具特色的现代田园城市。深入贯彻落实中央城市工作会议精神，进一步拓展发展空间，努力促进城乡统筹发展、区域协调发展、城乡一体化发展。持续推进综合交通枢纽建设。积极配合米字形快速铁路网建设，郑徐高铁年内开通运营，加快郑万、郑合高铁建设，尽快开工郑太、郑济高铁；开工建设高铁南站、机场至洛阳城际铁路。加快“畅通郑州”工程建设，农业快速路（除铁路代建部分）、京广快速路二期等工程建成通车，107辅道快速化工程（北三环至南三环部分）开工并实现高架主体贯通，加快西三环北延、北三环东延等工程建设，开工建设支线道路50条以上，建成通车30条以上；完成农村公路三年行动计划乡村通畅工程建设，持续完善县乡交通圈。加快公交都市建设，地铁2号线一期“十一”前试运营，1号线二期、南四环至机场城郊铁路年底前试运行，加快地铁5号线、3号线一期建设，扎实做好4号线、2号线二期、7号线、8号线、9号线前期工作，争取尽快开工；加快公交专用道、公交场站建设，新增新能源公交车500台以上。持续推进新型城镇化。力争6月底前围合区域内改造村庄完成拆迁，确保“四优先”村庄拆迁大头落地，力争2015年前拆迁村庄安置房全部开工建设。全面启动大围合区域内市场外迁工作，大力推进城区公益性农贸市场建设。积极做好历史文化风貌特色村和自然生态风貌特色村的保护提升。持续增强城市综合承载能力。加快侯寨水厂、双桥污水处理厂等一批水电气暖、污水及垃圾处理等市政设施建设，加快坚强智能电网建设；加快公共停车场、充换电站、充电桩、综合管廊和海绵城市建设，新增公共停车泊位5万个以上；注重区域协调发展，积极推进公共服务设施向城市薄弱地区和农村延伸。持续提升城市精细化管理服务水平。围绕打造“畅通、整洁、有序”市容环境、“天蓝、地绿、水清”生态环境和“15分钟便民生活圈”生活环境，加快“三级三类”便民服务中心建设，启动城市执法体制改革，实施提升县级城市管理水平三年行动计划，突出“四乱”治理，狠抓20项重点任务落实，提高城市精细化规范化数字化水平。特别是要按照“大中小修并重、有序推进大修、集中开展中小修”的原则，积极推进市政道路及附属设施整治。

（五）坚持绿色发展理念，大力推进生态文明建设。坚持源头严防、过程严管、后果严惩，齐抓共管，标本兼治，努力让良好生态环境成为人民生活质量的增长点。强力推进大气污染防治。紧紧抓住燃煤、扬尘、机动车尾气排放、散源排放四大污染源治理，以重点区域精细化治理为带动，力争市区禁燃区面积达到建成区面积的80%，中心城区机械化清扫冲洗率达到85%以上，市区建筑工地文明达标率达到100%；积极做好第五阶段标准车用汽柴油供应、机动车尾气治理、黄标车和老旧车辆淘汰、烟花爆竹禁放等工作；全面启动大围合区域工业企业外迁升级，三环内所有污染企业升级改造外迁或停产关闭；新力电力“热电外迁”工程全面开工建设，完成郑东新区热电厂、高新区泰祥电厂、荥阳国电等5家燃煤电厂11台机组超低排放改造。切实抓好水生态文明建设。加快推进贾鲁河生态水系综合整治工程，完成石佛沉砂池向西区供水工程、环城生态水系循环一期工程主体建设任务，开工建设牛口峪引黄工程；持续开展城市河流清洁行动，对十七里河、十八里河进行清淤，实施索须河（中州大道—祥云寺段）生态提升、潮河上游河道治理等工程建设；扎实做好“河长制”、“水长制”工作，努力实现“水清河美”目标。持续改善城乡生态环境。全面启动第十一届中国国际园林博览会园博园及配套设施建设，园博园主展馆力争年底前建成。大力开展植树造林和园林绿化，完成营造林10万亩，市区新增绿地500万平方米以上；加快环城高速林业生态景观带和森林公园体系建设，完成陇海西路、机场高速、京港澳高速等6条生态廊道建设任务；突出抓好南水北调生态文化公园、青少年公园、植物园二期等一批公园、游园建设，新建游园20个。大力推进“乡村清洁工程”，加强农村公路两侧“双违”整治，认真开展创建达标村和示范村活动，切实改善农村人居环境。着力加强生态环境管理。实施最严格的环境保护制度，严守各级政府环保责任红线，落实排污单位主体责任，实施一票否决。引入第三方治理，开展环境综合督查、重点领域执法检查，严厉打击环境违法行为。

（六）坚持共享发展理念，持续增强人民群众的现实获得感。继续加大财政投入，认真办理民生实事，切实让人民群众共享改革发展成果。全力打好脱贫攻坚战。深入贯彻精准扶贫基本方略，突出旅游引领、规划先行、分类实施、政府主导、社会化市场化推进，以易地扶贫搬迁为抓手，强力推进扶贫攻坚，实现脱贫3.2万人，荥阳市、新郑市实现扶贫对象全部脱贫，确保扶贫攻坚三年行动计划两年大头落地。积极推进创业就业。支持高校毕业生就业创业，做好退役军人和下岗职工安置工作，实施新生代农民工职业技能提升计划，突出抓好农村转移劳动力和城镇就业困难人员就业工作，发放小额担保贷款10亿元，新增城镇就业再就业13万人，农村劳动力转移就业7万人。加快发展教育事业。以实现均衡发展、促进教育公平为核心，进一步优化各阶段、各层次教育资源配置，制定鼓励社会资本参与教育基础设施建设的政策措施，市区新建改扩建中小学校30所，新增学位3万个。扎实推进健康郑州建设。持续加快宜居健康城和“十一中心”等一批医疗基础设施项目建设，新增床位1400张以上；完善公共卫生服务体系，推进基本公共卫生服务均等化。深入开展爱国卫生运动。落实全面两孩政策，提高出生人口素质。繁荣发展文化体育事业。创新发展文化产业，深入实施文化惠民和精品文化重点工程，加快建设“书香郑州”；强化网上思想文化阵地建设，打造繁荣清朗网络空间；加快推进市民公共文化服务区“四个中心”、大河村考古遗址公园等重点项目建设；认真筹办好丙申年黄帝故里拜祖大典、2016中国（郑州）国际旅游城市市长论坛和首届中国考古大会；深入开展全民健身运动，持续提升竞技体育水平。全面提高社会保障水平。扎实推进机关事业单位养老保险制度改革，深化“五险合一”，农村五保、城乡低保应保尽保；持续完善养老、优抚、助残、救孤、济困等制度，新建社区养老中心20个，新增养老床位2000张以上。健全农村留守老人、妇女、儿童关爱服务体系。支持慈善和红十字事业健康发展。切实加强社会治理。深化规范提升网格化管理，健全社会治安立体防控体系，完善反恐工作和打击犯罪机制，强化对流动人口和特殊人群的服务管理。畅通信访渠道，完善社会矛盾纠纷排查化解调处机制，切实做好信访稳定工作。加强社区建设，推行政府购买社区社会服务试点。落实属地管理责任和监管部门责任，集中解决重点地区、重点行业非法集资、融资担保等金融风险。加强食品药品安全监管，强化日常检查和督查抽检责任；严格落实安全生产责任制，深化重点领域专项整治，坚决遏制安全生产和食品药品及农产品安全重特大事故发生。加快平安郑州、法治郑州建设，确保社会大局和谐稳定。

加强国防动员、国防后备力量和人民防空建设，推动军民融合发展。支持驻郑解放军、武警部队建设，深化“双拥”共建工作。支持工、青、妇等人民团体更好发挥桥梁纽带作用。推进民族团结进步，促进宗教和谐和睦。全面做好统计、审计、地方志、外事侨务、对台事务、社科研究、档案管理、防震减灾等工作。

四、切实加强政府自身建设

全面贯彻法治政府建设实施纲要，加快推进法治政府、创新政府、廉洁政府和服务型政府建设。

（一）大力推进依法行政。健全依法决策机制，完善公众参与、专家论证、风险评估、合法性审查等重大决策法定程序。严格按照法定权限和程序行使权力，履行职责，推进政务公开。科学设计法治政府建设指标体系和考核标准，大力推进服务型行政执法，促进严格规范公正文明执法。加强重点领域立法，提高政府立法公众参与度。创新普法教育机制，大力推行“七五”普法。依法接受市人大及其常委会法律和工作监督，严格执行市人大及其常委会的决议决定，实行人大代表议案建议办理“一把手”负责制，规范办理程序和标准，提高办理质量和效率；主动接受市政协民主监督，切实做好政协委员提案办理工作，广泛听取社会各界意见，自觉接受社会监督和舆论监督。

（二）持续提升行政效能。规范四级政务服务体系，完善政务服务网功能，力争6月底前，实现便民服务网点覆盖率、政府信息资源共享率、已发布政务服务事项网上申报率“三个100%”；年底前，实现行政权责事项网上运行率、电子监察系统监管率、公共服务事项进驻大厅率“三个100%”。围绕实现“四个转变”和砍掉“五个一批”的目标，统筹推进行政审批、投资审批、公共资源交易制度等改革，着力解决跨领域、跨部门、跨层级的重大问题。

（三）加强政府作风建设。认真践行“三严三实”要求，全面贯彻中央八项规定精神，扎实开展“双学一做”学习教育，驰而不息纠正“四风”。加强公务员平时考核，实施公务员能力素质提升工程，深化争创人民满意公务员活动，提升公务人员素质能力。强化政府绩效管理和行政问责，持续开展懒政怠政为官不为专项治理，大力整治行政不作为、慢作为、乱作为现象。

（四）切实加强廉政建设。严明政治纪律和政治规矩，坚持纪严于法、纪在法前，认真履行主体责任和“一岗双责”，切实加强党风廉政建设，继续加大纠风惩腐力度，始终保持反腐高压态势。强化审计、监察等部门监督，严肃查处工程建设、土地出让、房地产开发、国有资产管理、矿产资源开发、公共资源交易等领域的腐败问题。加强对涉及群众切身利益的政府权力的监管，严肃处理涉及群众事务时吃拿卡要、与民争利甚至欺压群众的行为，以反腐倡廉的实效保障发展、取信于民。

各位代表：目标任务已经确立，路径重点已经清晰。时代在召唤，群众在期盼。让我们更加紧密团结在以习近平同志为总书记的党中央周围，在市委的坚强领导下，解放思想，振奋精神，埋头苦干，乘势而上，为实现“两个率先”目标、加快国际商都建设而努力奋斗！
